# Bioactive Cembrane Derivatives from the Indian Ocean Soft Coral, *Sinularia kavarattiensis*

**DOI:** 10.3390/md12074045

**Published:** 2014-07-03

**Authors:** Katja-Emilia Lillsunde, Carmen Festa, Harshada Adel, Simona De Marino, Valter Lombardi, Supriya Tilvi, Dorota A. Nawrot, Angela Zampella, Lisette D’Souza, Maria Valeria D’Auria, Päivi Tammela

**Affiliations:** 1 Centre for Drug Research, Division of Pharmaceutical Biosciences, Faculty of Pharmacy, P.O. Box 56, University of Helsinki, Helsinki FI-00014, Finland; E-Mails: katja-emilia.lillsunde@helsinki.fi (K.-E.L.); dorota.nawrot@helsinki.fi (D.A.N.); 2 Department of Pharmacy, University of Naples Federico II (USNF), Naples I-80131, Italy; E-Mails: carmen.festa@unina.it (C.F.); sidemari@unina.it (S.D.M.); azampell@unina.it (A.Z.); madauria@unina.it (M.V.D.); 3 CSIR-National Institute of Oceanography, Dona Paula, Goa 403004, India; E-Mails: hadel@nio.org (H.A.); supriyatilvi@nio.org (S.T.); lisette@nio.org (L.D.); 4 EuroEspes Biotechnology, Department of Cellular Immunology, Bergondo 15165, A Coruña, Spain; E-Mail: biotecnologia@ebiotec.com

**Keywords:** *Sinularia kavarattiensis*, soft coral, norcembranoid, NMR spectroscopy, Chikungunya, replicon cell line, live cell imaging, neuroinflammation

## Abstract

Marine organisms and their metabolites represent a unique source of potential pharmaceutical substances. In this study, we examined marine-derived substances for their bioactive properties in a cell-based Chikungunya virus (CHIKV) replicon model and for *in vitro* anti-inflammatory activity. In the screening of a marine sample library, crude extracts from the Indian soft coral, *Sinularia kavarattiensis*, showed promising activity against the CHIKV replicon. Bioassay-guided chemical fractionation of *S. kavarattiensis* resulted in the isolation of six known norcembranoids (**1**–**6**) and one new compound, named kavaranolide (**7**). The structures were elucidated on the basis of NMR and MS spectroscopic data. Compounds **1**–**3** and **5**–**7** were evaluated for their replicon-inhibiting potential in the CHIKV model by using a luminescence-based detection technique and live cell imaging. Compounds **1** and **2** showed moderate inhibition of the CHIKV replicon, but imaging studies also revealed cytotoxic properties. Moreover, the effects of the isolated compounds on primary microglial cells, an experimental model for neuroinflammation, were evaluated. Compound **2** was shown to modulate the immune response in microglial cells and to possess potential anti-inflammatory properties by dose-dependently reducing the release of pro- and anti-inflammatory cytokines.

## 1. Introduction

Chikungunya virus (CHIKV) is an alphavirus transmitted by *Aedes* mosquitoes and the cause of Chikungunya fever, a disease characterized by acute high fever, polyarthralgia, myalgia, nausea, headache and skin symptoms [1,2,3]. In addition to acute phase symptoms, CHIKV infection is often associated with chronic rheumatic manifestations that are relapsing and incapacitating [4,5]. The rheumatic symptoms can persist from months to years after the initial virus infection.

A new lineage of CHIKV emerged in 2004 as a result of a single mutation in the viral genome, which enabled the adaptation of the virus to the *Aedes albopictus* mosquito vector, a common vector of arthropod-borne diseases [6,7]. This mutation permitted the massive spread of the virus to many countries in the Indian Ocean region in the epidemics that escalated in 2005 [8]. As a consequence of vector adaptation and the resulting Chikungunya epidemic, local transmission of the virus has lately been reported not only in tropical, but also in temperate regions, such as Italy and south-eastern France [9,10,11]. There are currently neither vaccines nor specific therapies against CHIKV, and hence, infections can only be avoided by preventing exposure to mosquitoes in affected regions. The current treatment is symptomatic and mainly includes analgesics, anti-inflammatory drugs and corticosteroids [3].

The CHIKV genome is a single-stranded positive-sense RNA, which encodes for structural and non-structural proteins [12]. The genetic material is protected by a nucleocapsid, and the virion enters its host cell via receptor-mediated endocytosis. The drug discovery approaches applied to the treatment and prevention of CHIKV infection include entry inhibition, interference with viral protein translation, protein replication inhibition and modulation of the host immune response [13]. The replicon cell line used in our study expresses CHIKV non-structural proteins and can be used to identify potential CHIKV replication inhibitors. The replicon cell line enables safe and efficient screening that can be performed in a biosafety Level 2 laboratory. This is a great advantage compared to studies on infectious CHIKV, which requires handling in biosafety Level 3 facilities.

**Figure 1:**
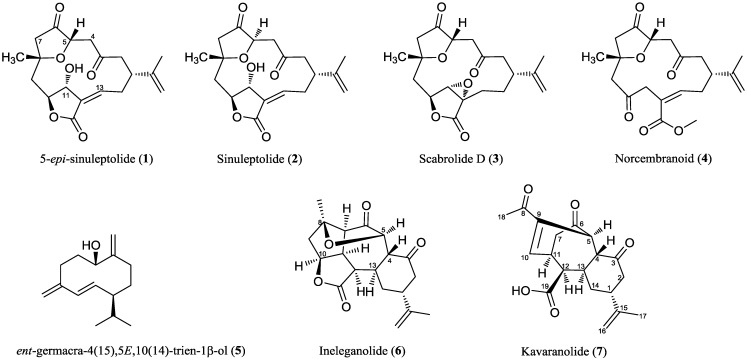
Compounds (**1**–**7**) isolated from the soft coral *S. kavarattiensis*.

In the course of the multinational collaborative project, MAREX, a library of extracts originating from marine organisms collected from the Indian Ocean were studied for their potential antiviral properties by using the CHIKV replicon model. The extract of the soft coral, *Sinularia kavarattiensis*, displayed promising anti-CHIKV activity and was consequently selected for bioassay-guided fractionation. The coral species of the genus, *Sinularia*, are widespread in coral reefs all over the world and have been reported to contain a variety of compounds with novel chemical structures [14]: sesquiterpenes [15,16], diterpenes [17], in particular cembranoids and norcembranoids, and polyhydroxylated steroids [18], which possess unique structural diversity. These metabolites display potential bioactivities, such as antimicrobial [14], anti-inflammatory [19,20,21,22,23,24], antiviral [25] and cytotoxic activity [26,27,28]. In the present study, the enriched chloroform extract of *S. kavarattiensis* afforded four known 14-membered macrocyclic norcembranoids, **1**–**4** (5-*epi*-sinuleptolide [29], sinuleptolide [30], scabrolide D [31,32] and norcembranoid **4** [33]), all of which lack a C-18 carbon substituent in their structures; one known germacrane-type sesquiterpenoid, **5** (*ent*-germacra-4(15),5*E*,10(14)-trien-1β-ol) [34], one known C_19_-norcembranoid diterpene ineleganolide, **6** [35], and the novel norcembranoid, named kavaranolide **7** (Figure 1), possessing a tricyclic carbocycle with the *trans*-fused six and seven-membered rings. 

*S. kavarattiensis* was primarily chosen for bioactivity-guided purification based on promising results against the CHIKV replicon. Corals of the genus, *Sinularia*, are, however, well-known sources of anti-inflammatory agents [19,20,21,22,23,24]. The isolated compounds were therefore, in addition to anti-CHIKV replicon activity, also studied for potential anti-inflammatory activity in primary microglial cells that serve as a model for neuroinflammation. Microglial cells are the resident immune cells of the central nervous system [36]. In the resting state, microglia are highly dynamic and control the environment by rapidly extending and retracting motile processes. Microglia are closely associated with astrocytes and neurons, particularly at the synapses, and many data indicate that neurotransmission plays a role in regulating the morphology and the function of surveying or resting microglia. The presence of reactive glia has been described in all neurodegenerative diseases, and microglial activation may contribute to the neuropathology observed [37,38]. The inhibition of neuroinflammation has been postulated as a putative target in the treatment of neurodegenerative diseases, and research has focused on the study of the potential neuroprotective effects of anti-inflammatory compounds in experimental models of neurodegeneration occurring in the presence of reactive glia.

The present paper describes bioassay-guided isolation, structure elucidation and antiviral and anti-inflammatory activity studies of the compounds isolated from *S. kavarattiensis*.

## 2. Results and Discussion

### 2.1. Bioassay-Guided Fractionation of S. kavarattiensis Extract

In a CHIKV replicon screen of a large marine sample library, we identified potential anti-CHIKV replication activity in the methanol and methanol-chloroform extracts from the marine soft coral, *S. kavarattiensis*. In primary screening, the extracts showed a 71% (methanol extract) and 72% (methanol-chloroform extract) decrease in the *Rluc* marker levels in the BHK-CHIKV-NCT (baby hamster kidney [BHK] cells expressing a non-cytotoxic [NCT] Chikungunya virus [CHIKV] replicon) cell line at a 100 μg/mL concentration. The crude extracts were tested for cytotoxic activity to rule out the possibility of the reduction in marker levels due to toxicity towards the host cell line. The extracts showed moderate cytotoxicity: the methanol extract caused 15% cytotoxicity, as measured by the reduction in ATP levels, and the methanol-chloroform extract a 17% reduction. As a follow up, the activity of the crude extracts was confirmed in dose-response assays. The extracts showed dose-dependent inhibition of the CHIKV replicon in the cell model. These promising results led to the selection of *S. kavarattiensis* for a bioactivity-guided purification study.

The crude methanol extract was fractionated according to the modified Kupchan partitioning procedure [39], and the obtained enriched extracts were studied for replicon inhibition and cytotoxic activity in the BHK-CHIKV-NCT cell line. At a 100 μg/mL concentration, the chloroform- and ethyl acetate-enriched extracts proved to possess inhibitory activity against the CHIKV replicon, causing a 47% and 65% decrease of the *Rluc* marker level, with a cytotoxic effect of 24% and 20%, respectively. The chloroform-enriched extract (3.2 g) was further fractionated by silica gel MPLC using a solvent gradient system from CH_2_Cl_2_ to MeOH followed by reverse phase HPLC to afford pure compounds.

### 2.2. Chemical Characterization

Kavaranolide **7** was isolated as a white amorphous solid, and its formula of C_19_H_22_O_5_, implying nine degrees of unsaturation, was established by high-resolution ESIMS based on the pseudomolecular ion [M − H]^−^ at *m*/*z* 329.1381.

The ^13^C NMR data confirmed the presence of 19 carbons (Table 1), including three ketone signals at δ_C_ 195.7, 207.9 and 211.3, one acyl signal at δ_C_ 174.1, one trisubstituted double bond (δ_C_ 149.0, d and 139.5, s) and one disubstituted double bond (δ_C_ 147.2, s and 111.2, t). The carbonyl and olefinic carbons account for six degrees of unsaturation; hence, the compound is tricyclic. Analysis of the ^1^H NMR spectrum revealed an isopropenyl group (δ_H_ 1.63 (3H, s), 4.43, (1H, br s) and 4.75 (1H, br s)), a deshielded olefin proton at δ_H_ 7.36 (1H, br d, *J* = 7.5 Hz) and a ketone methyl at δ_H_ 2.21 (3H, s).

**Figure 2:**
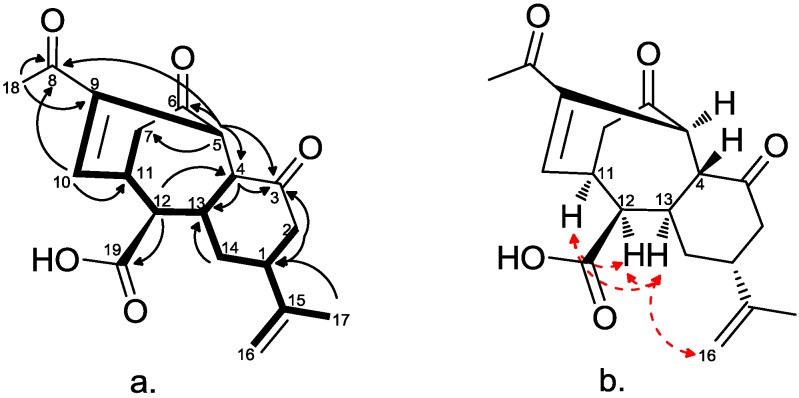
(**a**) COSY connectivities (bold bonds) and key HMBC (black arrows) correlations; (**b**) key NOE (red dashed arrows) correlations for Compound **7**.

Careful analysis of the COSY spectrum allowed us to build up a single spin system (Figure 2): H-5, H-10 (by allylic coupling), H-11 (H_2_-7), H-12, H-13 (H-4), H_2_-14, H-1 (H_2_-16 and H_3_-17, by allylic coupling), H_2_-2. Even if no scalar coupling was observed between H-4 and H-5, the linkage C-4/C-5, as well as the location of two ketone functions at C-3 and C-6 were easily inferred from HMBC correlations H-5/C-3, C-4, C-6, C-7 and H-4/C-3, C-5, C-6, C-13. The acetyl group was placed at C-9 on the basis of HMBC correlations H_3_-18/C-8, C-9, whereas the long-range correlation, H-12/C-19, allowed the placing of the carboxy group at C-12. These data, together with additional HMBC correlations (Table 1 and Figure 2) defined the planar structure of kavaranolide (**7**), as depicted in Figure 1.

**Table 1:** ^1^H and ^13^C NMR data (700 and 175 MHz, DMSO-*d*_6_) of kavaranolide (**7**).

	δ_H_^a^	δ_C_	HMBC
1	2.62 m	39.9	
2α	2.50 ^b^	43.9	C1, C3, C15
2β	2.36 m		C3, C14
3	-	207.9	
4	3.12 d (12.6)	50.5	C3, C5, C6, C13
5	4.30 br s	43.3	C3, C4, C6, C7, C8, C9, C10, C13
6	-	211.3	
7α	2.19 ^c^	39.2	C6, C10, C11, C12
7β	2.24 m		C6, C12
8	-	195.7	
9	-	139.5	
10	7.36 br d (7.5)	149.0	C5, C7, C8, C11
11	3.17 m	34.7	
12	2.29 dd (5.2, 7.5)	48.8	C4, C10, C13, C19
13	1.84 m	36.1	
14α	1.55 m	31.7	
14β	2.44 m		C13, C15
15	-	147.2	
16	4.75 br s	111.2	C1, C15, C17
	4.43 br s		C1, C17
17	1.63 s	21.8	C1, C15, C16
18	2.21 s	24.8	C8, C9
19	-	174.1	

^a^ Coupling constants are in parentheses and given in hertz. ^1^H and ^13^C assignments were aided by COSY, HSQC and HMBC experiments; ^b^ overlapped with the solvent signal; ^c^ overlapped with the other signal.

The relative stereochemistry of Compound **7** was deduced from the analysis of the coupling constants and some key dipolar coupling evidenced by the NOESY spectrum (Figure 2).

The NOE correlations between H-13/H_2_-16, H-11 and H-12 suggested that they are on the same face of the rings. By analogy with ineleganolide [35], which co-occurs in the same specimen and for which the relative configuration has been established by X-ray diffraction analysis, we assigned these signals as α protons.

The six- and seven-membered rings are *trans*-fused on the basis of the large value of *J*_H-4/H-13_ (12.6 Hz); therefore, H-4 has a β-orientation. When it comes to the H-5 configuration, no diagnostic dipolar couplings were observed for this proton. The absence of scalar couplings between H-4 and H-5 indicated that they have a dihedral angle of *ca.* 90°. Force field calculations evidenced that the closure of the bridged structure of kavaranolide and the geometric relationship between H-4 and H-5 were only compatible with an α-orientation of the H-5 proton. Therefore, the stereostructure of kavaranolide (**7**) was determined as depicted in Figure 1. The bridged 6,7,6-ring-fused framework of kavaranolide closely resembles that of ineleganolide (**6**), also isolated from the extract of *S. kavarattiensis* in the present study, and of horiolide (**11**), isolated from an Indian Ocean collection of *Sinularia* sp. [40]. The similar experimental scalar and dipolar coupling patterns observed for ineleganolide (**6**), horiolide (**11**) and kavaranolide (**7**) gave further support to the structural assignment. Furthermore, the observed structural and stereochemical homology between these derivatives suggested a common biosynthetic origin.

**Scheme 1:**
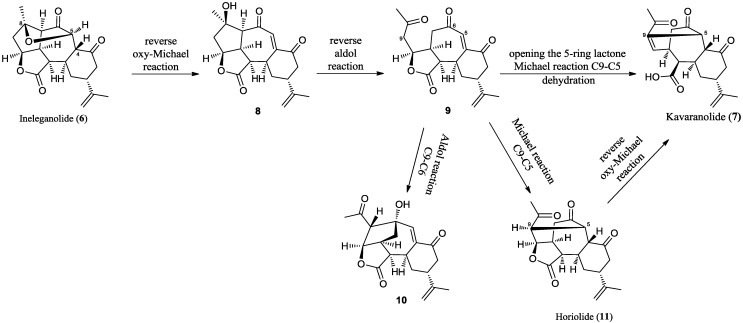
A plausible biosynthetic route to kavaranolide (**7**).

In a recent report, Li *et al.* [41] proposed biosynthetic pathways that likely lead to polycyclic skeletons of norcembranoids through transannular Michael reactions from norcembranoid macrocyclic precursors. In particular, they speculated that the tricyclic ring system in ineleganolide (**6**) may originate *in vivo* from 5-*epi*-sinuleptolide (**1**) by two successive Michael reactions, involving the nucleophilic centers at C-4 and C-7 and the electrophilic centers at C-13 and C-11 in **1** [41]. The proposed biosynthetic route to **6** from **1** was strongly supported by subsequent studies, which demonstrated the *in vitro* conversion of **1** to **6**, in strong base conditions [42]. Interestingly it turned out that under different experimental conditions, the main product was the novel polycyclic derivative, **10** (Scheme 1). Its formation from ineleganolide (**6**) was proposed to take place by a reverse oxy-Michael reaction and two successive aldol reactions. The key trione intermediate, **9**, could undergo an aldol reaction leading to the polycyclic derivative, **10**, or a Michael reaction leading to horiolide (**11**). In this context, kavaranolide (**7**) is likely to be derived *in vivo* from horiolide (**11**) by a reverse oxy-Michael reaction (Scheme 1). It is also conceivable that kavaranolide (**7**) could be derived from trione, **9**, by opening of the 5-ring lactone followed by an intramolecular Michael reaction and then dehydration. As neither intermediate **9** nor intermediate **10** have been reported as natural compounds, the isolation of kavaranolide (**7**) gave further support to the proposed biosynthetic route.

### 2.3. Inhibition of CHIKV Replicon by Compounds Isolated from S. kavarattiensis

#### 2.3.1. Primary Evaluation by *Rluc* Detection and ATP Quantitation

**Figure 3:**
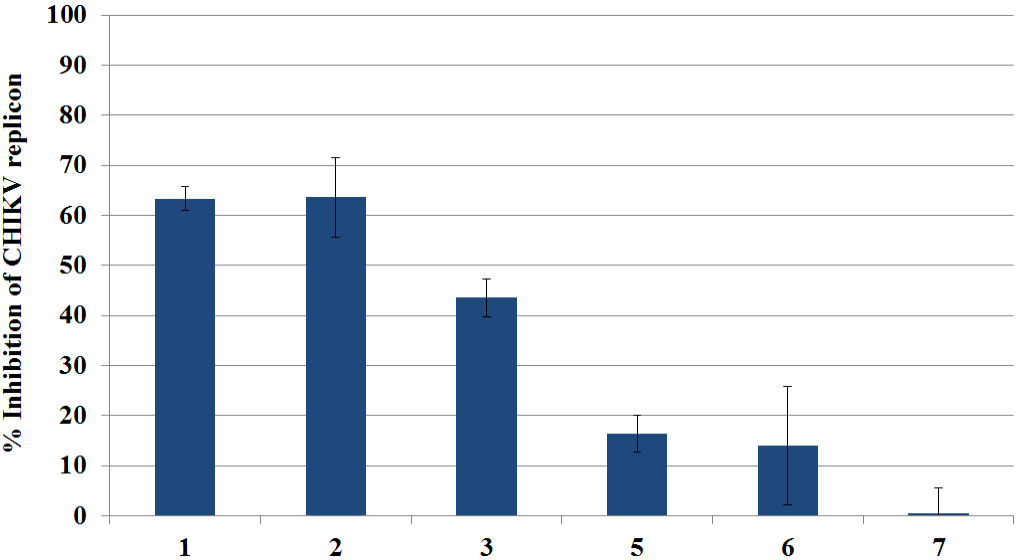
Inhibition of the Chikungunya virus (CHIKV) replicon in the primary evaluation of pure Compounds **1**–**3** and **5**–**7**, determined by the measurement of *Rluc* marker levels at a 100 μM concentration. The percentage of inhibition is based on a comparison of the test compound *Rluc* levels to the DMSO vehicle control. Inhibition percentages are the averages of the results from three test wells; error bars represent the standard deviation. The positive control, 6-azauridine, inhibited the replicon with an average IC_50_ value of 2 μM.

The replicon-inhibiting potential of Compounds **1**–**3** and **5**–**7** was primarily evaluated in the BHK-CHIKV-NCT cell line by detection of *Rluc* marker levels as a measure of the inhibition of the CHIKV replicon. Additionally, we evaluated the compounds for cytotoxic activity by ATP quantitation. The compounds, tested at a 100 μM concentration, showed the inhibition of the CHIKV replicon ranging from 0.5% to 63% (Figure 3). The strongest inhibitory effects were observed for Compounds **1** and **2**, which both inhibited the CHIKV replicon by more than 60% compared to the vehicle control. All compounds showed none or only very slight cytotoxicity (<6%) in the ATP assay.

#### 2.3.2. Live Cell Imaging

**Figure 4:**
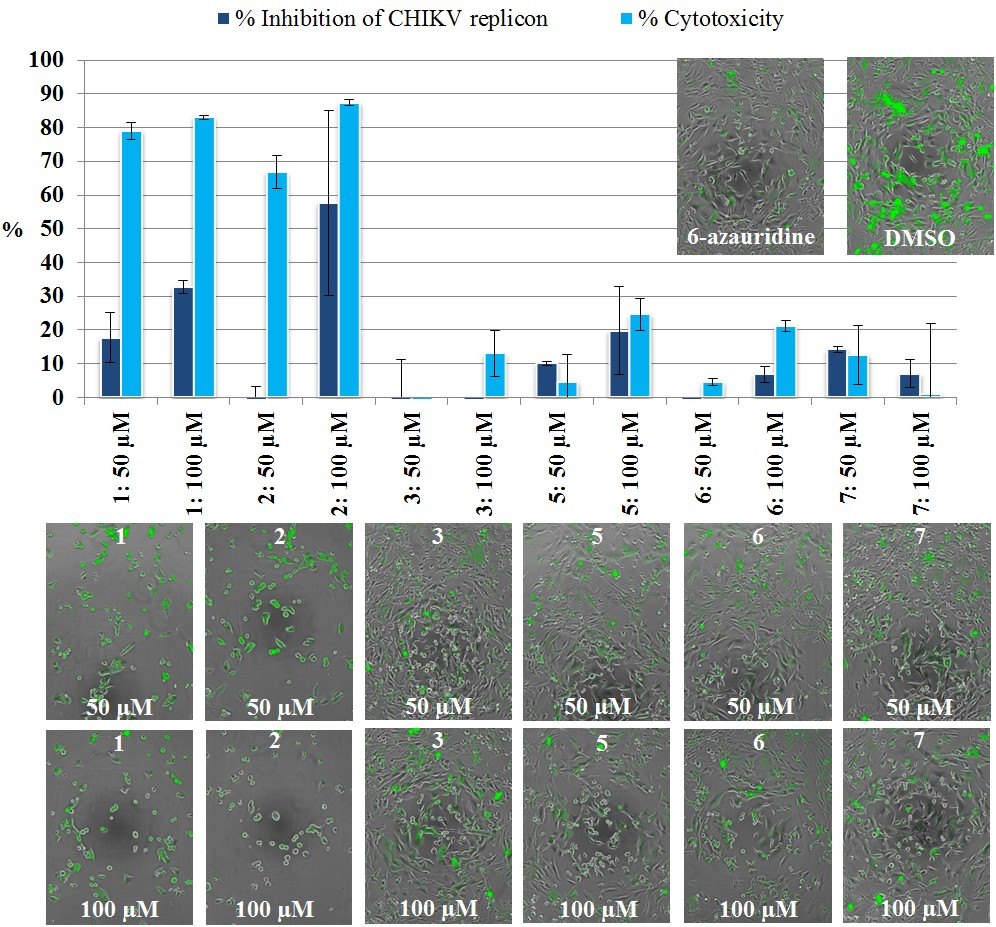
The inhibition of the CHIKV replicon and the cytotoxicity of Compounds **1**–**3** and **5**–**7** measured by Cell-IQ^®^ live cell imaging in the BHK-CHIKV-NCT cell line at test concentrations of 50 and 100 μM. Results are shown together with representative photomicrographs captured after 48 h. The percentage of inhibition and cytotoxicity were calculated by a comparison to the DMSO vehicle control. Values are the averages of two independent assays with three replicates each; error bars represent the standard deviation. Pictomicrographs of cells treated with control compounds (10 μM 6-azauridine and 0.5% DMSO) for 48 h are shown for comparison. More detailed information on the effects of control compounds in the live cell imaging study can be found in Chapter 3.5.4.

As a follow-up study to the primary assay based on the determination of *Rluc* expression, cells treated with Compounds **1**–**3** and **5**–**7** were monitored for 48 h by live cell imaging in the Cell-IQ^®^ platform. The expression of EGFP marker levels was followed up through fluorescence intensity measurements. This confirmed that Compounds **1** and **2** cause the strongest decrease in marker protein levels (Figure 4), as observed previously by *Rluc* detection. Compounds **5**, **6** and **7** caused only moderate decreases in the fluorescence intensity. Compound **3**, which at a 100 μM concentration reduced *Rluc* marker levels by 44%, turned out to be inactive in the Cell-IQ^®^ experiments at both test concentrations of 50 and 100 μM. Furthermore, the fluorescence inhibition of **1** was not as prominent as the *Rluc* inhibition previously observed (Figure 3).

Furthermore, the Cell-IQ^®^ experiment revealed the cytotoxic effects of Compounds **1** and **2**, which were not observed by ATP quantitation. At a 100 μM concentration, Compounds **1** and **2** decreased the amount of viable cells (cells classified by the Cell-IQ Analyser^®^ as living or dividing) by >80%, whereas the effects on the fluorescence intensity levels were not as drastic. The reasons behind these observations remain unclear; however, we hypothesize that the relatively high test concentrations provoking the cytotoxic properties of Compounds **1** and **2** may lie behind the inconsistencies. In order to further clarify the reasons behind the decrease in cell viability caused by Compounds **1** and **2**, the ATP quantitation and imaging assays were repeated by using the same cell concentration (3000 cells/well) in both experiments. At this concentration, the cell population reaches confluence in 48–72 h, as opposed to the standard concentration of 40,000 cells/well used in the ATP assay, where confluence is reached after overnight incubation. The lower cell concentration applied in live cell imaging facilitates cell classification and the observation of changes in the morphology throughout the experiment. These additional experiments revealed that the cell concentration has a major impact on the resistance of the BHK-CHIKV-NCT cells to the cytotoxic effects of Compounds **1** and **2**. Indeed, when the cell concentration was lowered to 3000 cells/well, Compound **1** reduced the ATP level by 39% and **2** by 44% at a 100 μM concentration. These results are supported by the fact that cell confluence in general is known to affect cellular responses and, thereby, results in live cell imaging studies [43].

The results from the imaging study also imply that the test concentration may impact the mode of action of Compounds **1** and **2**. At a 50 μM concentration of **1** and **2**, the cell number decreased drastically during the 48 h test, but the remaining cells still appeared viable and bright green in fluorescence microscopy images. At a 100 μM concentration, the cell number likewise decreased, and furthermore, the remaining cells were rounded in shape with the reduced expression of EGFP. Based on the results from our study and previous studies on the cytotoxic properties of **1** and **2** [44,45], we hypothesize that at a concentration of 100 μM, the apoptotic effects are prevailing. At the lower concentration of 50 μM, the reduced number of viable cells did surprisingly not have a major influence on the overall fluorescence intensity.

The results obtained for the model antiviral compound, 6-azauridine, and the cytotoxic control compound, polymyxin B, in the *Rluc* detection and ATP assays on BHK-CHIKV-NCT cells corresponded to the results obtained in Cell-IQ^®^. Therefore, the control compounds were used to confirm assay accuracy in Cell-IQ^®^ and to validate the new detection method that the imaging platform offers (see Chapter 3.5.4 for more details). As demonstrated in Figure 4, the inhibition of the CHIKV replicon is mainly achieved at a 100 μM concentration of Compounds **1** and **2**, which clearly also triggers cytotoxicity. The predominance of cytotoxic effects at high concentrations likewise makes further investigation of the dose-response correlation in the CHIKV replicon model challenging and uncertain. For these reasons, the determination of IC_50_-values for these compounds was not meaningful. The results obtained for Compounds **1** and **2** by means of live cell imaging and ATP quantitation accentuate the importance of these follow-up studies in the thorough investigation of compound properties in the cell model, before proceeding to more complex and risky experiments, such as studies on infectious viruses. Furthermore, Cell-IQ^®^ enables constant visual monitoring of the cells, as well as their expression of the EGFP marker, whereas *Rluc* detection and ATP quantitation give only end-point data without the possibility to visualize the results.

We can summarize that imaging in Cell-IQ^®^ completes the information obtained by the primary detection techniques. In the present study, the imaging studies guided us to a more thorough investigation of the cytotoxic properties of the compounds obtained from *S. kavarattiensis*. As a conclusion, a marked CHIKV replicon inhibitory potential was found for the crude extracts of *S. kavarattiensis*, whereas Compounds **1** and **2** exhibited moderate inhibition of the CHIKV replicon along with prevailing cytotoxic activity.

### 2.4. Anti-Inflammatory Activity of Isolated Compounds

**Figure 5:**
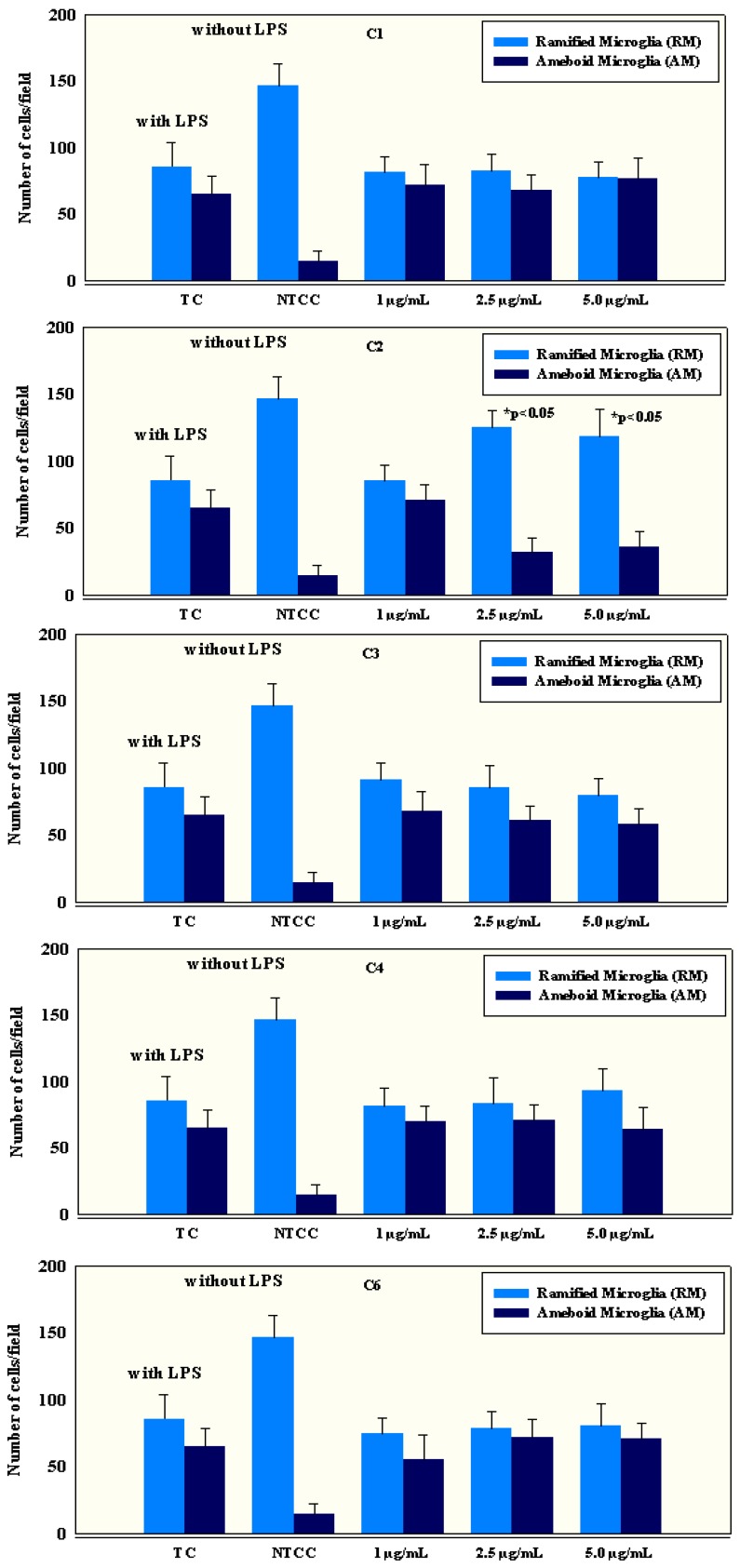
The effects on primary microglial cells in LPS-treated cultures (TC), non-treated cell cultures (NTCC) and after stimulation of LPS-treated cultures with different concentrations (1 μg/mL, 2.5 μg/mL and 5 μg/mL) of Compounds **1**–**4** and **6**. In the healthy CNS, microglial cells have a highly ramified morphology with thin processes. Microglia, in this surveillance state, have been defined as ramified microglia. In contrast, reactive microglia (*i.e.*, microglia that are no longer ramified microglia) can adopt different morphologies, including a hypertrophic cell with enlarged processes or an amoeboid macrophage-like morphology. Microglial cell density quantification (number of cells/field) in the treated and untreated primary microglial cell cultures shows the ability of Compound **2** at 2.5 μg/mL and 5.0 μg/mL to significantly reduce the number of activated amoeboid cells (*p* < 0.05 with respect to the TC control) and increase the number of ramified/resting cells (*p* < 0.05 with respect to the TC control).

The biological activity of isolated Compounds **1**–**4** and **6** was tested by using primary microglial cell cultures. Microglial cells in untreated cell cultures usually appeared as ramified cells homogeneously distributed in the cultures. These ramified microglial (RM) cells, resembling the ramified type of microglia *in vivo*, displayed a pleomorphic cell body with a variable number of branching processes, sometimes with a spiny-like appearance. The cells were usually found as isolated cells without forming cell clusters. Apart from the RM, a number of amoeboid microglia (AM) was also distinguished. AM were smaller than RM and displayed pseudopodia and/or filopodia instead of cell processes. The AM cell density in non-treated cell cultures (NTCC) was about 85% lower than in lipopolysaccharide (LPS)-treated cultures (TC). After treatment with three different concentrations (1.0 μg/mL; 2.5 μg/mL and 5.0 μg/mL) of Compounds **1**–**4** and **6**, significant RM and AM mean differences were observed in cultures treated with 2.5 μg/mL (7.2 μM) and 5.0 μg/mL (14.4 μM) of **2** (Figure 5). The observed results suggest that **2** was able to induce a shift from AM to RM shapes. Since AM cells are mainly activated cells, we conclude that this compound shows the ability to modulate an immune response and could interfere with the progression of chronic neurodegenerative diseases, although the underlying mechanisms are still unclear.

Microglia have the capacity to release a large number of substances that can be detrimental to the surrounding neurons, including glutamate, ATP, reactive oxygen species and pro-inflammatory cytokines. In glial cultures, different pro- and anti-inflammatory cytokines, as well as reactive oxygen and nitrogen species, are produced in response to LPS treatment. These factors are involved in the inflammatory response of reactive glial cells and the resolution of the inflammation. IL-10 is a cytokine with main immunoregulatory and anti-inflammatory properties. Several studies have shown the inhibitory effect of IL-10 treatment on the production of pro-inflammatory cytokines by reactive glia in response to LPS [46,47]. In addition to the morphological changes, cytokine release in supernatants was also quantified both in treated and control microglial cultures. The levels of pro-inflammatory cytokines, such as IL-1β, IL-6, IL-8, IL-18 and TNF-α, were significantly (*p* < 0.05) elevated (Table 2) in primary microglial cultures treated with LPS compared to controls. A significant difference was observed in the levels of pro-inflammatory cytokines IL-1β, IL-6, IL-8, IL-18, TNF-α, as well as in anti-inflammatory cytokines, such as IL-4 and IL-10, between Compound **2** at concentrations of 2.5 μg/mL (7.2 μM) and 5.0 μg/mL (14.4 μM) and the controls (Table 2).

**Table 2:** Cytokine release (pg/mL) in the supernatants of primary microglial cells after 72 h of stimulation with different concentrations (C1: 1.0 μg/mL; C2: 2.5 μg/mL; and C3: 5.0 μg/mL) of Compounds **1**–**4** and **6**.

	IL-1β	IL-6	IL-8	IL-18	TNF-α	IL-4	IL-10
**LPS-treated (TC)** (1 μg/mL)	1.05 ± 0.06	13.5 ± 1.2	257 ± 5.7	325 ± 8.4	34.5 ± 12.2	1.4 ± 2.4	1.6 ± 2.1
**Non-treated (NTCC)** (Medium)	0.05 ± 0.02	1.5 ± 0.8	7 ± 1.1	8 ± 1.3	5 ± 1.8	7.3 ± 0.05	8.1 ± 0.08
**1**(C1)	1.04 ± 0.08	14.2 ± 1.8	256 ± 12	319 ± 8.1	33.5 ± 11.2	1.9 ± 0.9	7.6 ± 1.6
**1**(C2)	1.09 ± 0.1	11.7 ± 1.2	239 ± 5	318 ± 7.5	31.2 ± 9.2	2.1 ± 0.8	8.2 ± 1.8
**1**(C3)	1.08 ± 0.05	13.5 ± 1.4	241 ± 7	315 ± 8.1	33.6 ± 11.1	1.9 ± 0.4	8.7 ± 2.1
**2**(C1)	1.08 ± 0.02	12.1 ± 0.9	249±13	326 ± 4.9	34.1 ± 9.1	2.1 ± 0.9	7.9 ± 3.4
**2**(C2)	0.07 ± 0.04 *	7.8 ± 1.4 *	123 ± 11 *	187 ± 14 *	12.3 ± 3.4 *	15.5 ± 2.3 *	17.4 ± 2.3 *
**2**(C3)	0.08 ± 0.06 *	8.5 ± 1.3 *	108 ± 9 *	155 ± 11 *	9.8 ± 3.3 *	18.4 ± 1.8 *	18.5 ± 1.9 *
**3**(C1)	1.1 ± 0.05	12.9 ± 1.1	247 ± 8	312 ± 8.2	33.6 ± 11.2	1.5 ± 0.9	7.5 ± 1.4
**3**(C2)	1.3 ± 0.06	11.5 ± 1.3	237 ± 4	321 ± 7.8	24.5 ± 6.9	1.6 ± 0.8	6.8 ± 1.5
**3**(C3)	1.2 ± 0.05	13.2 ± 1.2	244 ± 11	318 ± 7.6	35.4 ± 12.4	1.7 ± 0.5	6.9 ± 1.5
**4**(C1)	1.01 ± 0.04	12.1 ± 1.3	247 ± 8	312 ± 8.2	33.2 ± 2.8	2.2 ± 0.2	8.4 ± 2.6
**4**(C2)	1.02 ± 0.04	14.1 ± 1.1	247 ± 8	312 ± 8.2	29.8 ± 2.5	2.3 ± 0.5	7.9 ± 2.5
**4**(C3)	1.01 ± 0.02	12.1 ± 1.3	247 ± 8	312 ± 8.2	32.5 ± 3.7	1.8 ± 0.2	7.8 ± 3.2
**6**(C1)	1.07 ± 0.07	13.2 ± 0.2	247 ± 8	312 ± 8.2	31.2 ± 2.9	1.8 ± 0.6	8.5 ± 3.2
**6**(C2)	1.1 ± 0.05	12.1 ± 0.9	247 ± 8	312 ± 8.2	30.6 ± 2.6	1.9 ± 0.6	7.8 ± 2.2
**6**(C3)	1.09 ± 0.02	11.8 ± 1.2	247 ± 8	312 ± 8.2	32.3 ± 3.2	1.9 ± 0.9	7.5 ± 2.1

* *p* < 0.05.

**Table 3:** *In vitro* cytotoxic effects (IC_50_) on primary microglial cell cultures and the inhibitory effect on LPS-induced pro-inflammatory cytokine release of Compounds **1**–**4** and **6**. Each value represents the mean of triplicate determinations.

Sample	Cytotoxicity	IL-1β	IL-6	IL-8	IL-18	TNF-α	IL-4	IL-10
IC_50_ (μg/mL)								
**1**	>100	>12.5	>12.5	>12.5	>12.5	>12.5	>12.5	>12.5
**2**	>100	**0.5**	**2.2**	**2.3**	**2.9**	**1.7**	**0.5**	**0.5**
**3**	>100	>12.5	>12.5	>12.5	>12.5	>12.5	>12.5	>12.5
**4**	>100	>12.5	>12.5	>12.5	>12.5	>12.5	>12.5	>12.5
**6**	>100	>12.5	>12.5	>12.5	>12.5	>12.5	>12.5	>12.5
Tamoxifen	3.60	ND	ND	ND	ND	ND	ND	ND
Prednisolone	ND	12	13	15	16	10	20	15

ND: not determined.

Furthermore, the inhibition of cytokine release and the cytotoxicity towards primary microglial cell cultures was measured at several compound concentrations to determine the IC_50_ values (Table 3). Compounds **1**–**4** and **6** were tested for cytotoxicity at seven concentrations ranging from 1.56 to 100 μg/mL and for cytokine release at six concentrations ranging from 0.39 to 12.5 μg/mL. Tamoxifen and prednisolone were used as positive controls in the MTT assay and in cytokine quantification, respectively. Similarly to previous data presented in Table 2, Compound **2** showed effective and dose-dependent inhibition of cytokine release. As demonstrated in Table 3, the IC_50_-values for the inhibition of cytokine release for Compound **2** ranged from 0.5 μg/mL (1.4 μM) to 2.9 μg/mL (8.3 μM); all of them being markedly lower than those of the positive control prednisolone. The IC_50_-values for cytotoxic activity for each of the tested compounds, **1**–**4** and **6**, turned out to be higher than the maximal test concentration of 100 μg/mL, and the cytotoxicity was thus not influencing the observed anti-inflammatory properties. In accordance with these results, we suggest that among new anti-inflammatory agents, special attention should be paid to Compound **2**.

### 2.5. Bioactivity Potential and Structure-Activity Relationships of Metabolites from S. kavarattiensis

The anti-inflammatory properties of metabolites from *Sinularia* species have previously been reported in numerous studies [19,20,21,22,23,24]. Our results imply that Compound **2**, in addition to anti-inflammatory activity, also possesses promising immunomodulatory activity. Takaki and coworkers described the anti-inflammatory effects of **2** by demonstrating the inhibition of LPS-induced TNF-α production in murine macrophage-like cells [48]. Our results show that the anti-inflammatory activity of **2**, except for the inhibition of TNF-α release, also takes place through the inhibition of the release of cytokines that belong to the interleukin family. 

The CHIKV replicon-inhibiting potential of isolated Compounds **1**–**3** and **5**–**7** was not as striking as the activity observed for the crude extracts and the enriched fractions of *S. kavarattiensis*. On the one hand, the reason behind this observation may be the synergetic activity of the compounds in the extracts and fractions, which leads to higher inhibition percentages than those exhibited by each compound separately. On the other hand, the primary test concentration for the extracts and fractions was as high as 100 μg/mL, whereas the test concentration of the pure compounds in the CHIKV replicon model, 100 μM, equals a concentration of 35 μg/mL in the case of Compounds **1** and **2**.

5-*epi*-Sinuleptolide **1** and sinuleptolide **2** share a common α-β-unsaturated-γ-butyrolactone moiety, which is recognized to play a pivotal role in the interactions with unidentified molecular targets [49,50]. The inactivity in both the CHIKV replicon model and the anti-inflammatory assays of Compounds **3**–**7** that lack this functionality strongly suggests the involvement of an electrophilic conjugated function in **1** and **2**, which could act as a Michael acceptor toward reactive lysine or cysteine residues in the biological targets. Compound **1** and **2** only differ from each other regarding the configuration of one stereogenic center. The inactivity in the anti-inflammatory assays of Compound **1** is, however, not surprising. The covalent interaction with protein targets requires a correct positioning through specific the non-covalent interactions of the active functional group within the active site of the biological target.

The observed cytotoxicity against the BHK-CHIKV-NCT at high concentrations of Compounds **1** and **2** could be explained in terms of their potential Michael acceptor activity, which can lead to cell damage and general toxicity [51]. The predominating cytotoxic properties at the effective concentrations make further exploration of the antiviral potential of these compounds challenging. On the contrary, Compound **2** inhibited the release of pro-inflammatory cytokines and displayed immunomodulatory activity at concentrations that caused no cytotoxicity against the primary microglial cells used in our study. Huang and coworkers [26] have described the cytotoxic properties of the acetate of **1** (5-*epi*-sinuleptolide acetate) at low concentrations against a panel of cancer cell lines, demonstrating apoptosis induction and cell cycle arrest to lie behind the cytotoxic effects. Furthermore, Liang and coworkers has demonstrated that **1** triggers cell cycle arrest and apoptosis in skin cancer cells at low concentrations [44]. Liang *et al.* studied the cytotoxicity of both **1** and **2** and found that **1** causes more prominent cytotoxic effects with lower IC_50_-values against the studied cancer cell lines. Our results imply that at 50 μM concentration, **1** is slightly more cytotoxic to the BHK-CHIKV-NCT cells than **2**; however, at 100 μM, the differences in cytotoxicity are minor. The impact of cellular confluence is nevertheless critical. Our results show that cell cultures that have reached confluence before the treatment with compounds is started are more resistant to the cytotoxic effects.

## 3. Experimental Section

### 3.1. General Experimental Procedures

Specific rotations were measured on a PerkinElmer 243 B polarimeter. High-resolution ESI-MS spectra were performed with a Micromass QTOF Micromass spectrometer. ESI-MS experiments were performed on an Applied Biosystem API 2000 triple-quadrupole mass spectrometer. NMR spectra were obtained on Varian Inova 700 MHz spectrometer (^1^H at 700 MHz, ^13^C at 175 MHz, respectively) equipped with a Sun hardware, δ (ppm), *J* in Hz; and spectra referred to DMSO-*d*_6_ (δ_H_ 2.50, δ_C_ 39.5) as the internal standard. Through-space ^1^H connectivities were evidenced using a ROESY experiment with a mixing time of 200 and 300 ms. HPLC was performed using a Waters Model 510 pump equipped with a Waters Rheodyne injector and a differential refractometer, model 401. Silica gel (200–400 mesh) from Macherey-Nagel Company (Düren, Germany) was used for flash chromatography. The purities of compounds were determined to be greater than 95% by HPLC.

### 3.2. Biological Material

Soft coral, *Sinularia kavarattiensis* Alderslade & Prita, was collected off the coast of Rameshwaram, Tamil Nadu, India (Latitude: 9°16′60′′N Longitude: 79°17′60′′E) in December 2010. It was frozen at −20 °C and transferred to the Council of Scientific and Industrial Research-National Institute of Oceanography (CSIR-NIO) Laboratory, Goa, India. The organism was identified by Panachamoottil Abraham Thomas, Emeritus Scientist, Vizhingam Research Center, Central Marine Fisheries Research Institute, Kerala, India. A voucher specimen (14S021) is deposited at the CSIR-NIO. Four hundred grams of freeze-dried organism were extracted four times with 80% methanol (500 mL) each time to obtain 23 g of the crude methanolic extract. The same sample was then extracted three times with methanol:chloroform (1:1). The extracts were concentrated at 30 °C using a Rotavapor and a vacuum pump.

### 3.3. Chemical Characterization

The crude methanolic extract (18.061 g) was subjected to a modified Kupchan’s partitioning procedure [39], as follows. The methanol extract was dissolved in a mixture of MeOH/H_2_O containing 10% H_2_O and partitioned against *n*-hexane to give 2.9 g of the crude extract. The water content (% *v/v*) of the MeOH extract was adjusted to 30% and partitioned against CHCl_3_ to give 3.2 g of the crude extract. The aqueous phase was concentrated to remove MeOH and, then, subsequently extracted with ethyl acetate (0.29 g) and with *n*-BuOH (1.6 g).

The CHCl_3_ extract (3.2 g) was fractionated by silica gel MPLC using a solvent gradient system from CH_2_Cl_2_ to MeOH. The fraction eluted with CH_2_Cl_2_/MeOH 995:5 (138 mg) contained pure *ent*-germacra-4(15),5*E*,10(14)-trien-1β-ol **5** (19 mg; 0.005% yield). The fraction eluted with CH_2_Cl_2_/MeOH 99:1 (101 mg) was further purified by HPLC on a Nucleodur 100-5 C18 (5 μm; 4.6 mm i.d. × 250 mm) with 55% MeOH/H_2_O as the eluent (flow rate 1 mL/min) to give 1.3 mg of kavaranolide **7** (*t*_R_ = 8.5 min, 0.0003% yield), 3.9 mg of ineleganolide **6** (*t*_R_ = 10 min; 0.001% yield), 2.4 mg of scabrolide D **3** (*t*_R_ = 15.5 min; 0.0006% yield) and 1.0 mg of norcembranoid **4** (*t*_R_ = 19.5 min; 0.0003% yield). The other fraction eluted with CH_2_Cl_2_/MeOH 99:1 (125 mg) was further purified by HPLC on a Nucleodur 100-5 C18 (5 μm; 4.6 mm i.d. × 250 mm) with 40% MeOH/H_2_O as the eluent (flow rate 1 mL/min) to give 13.0 mg of 5-*epi*-sinuleptolide **1** (*t*_R_ = 20 min; 0.003% yield) and 8.0 mg of sinuleptolide **2** (*t*_R_ = 24 min; 0.002% yield). The reported percentage yields are referred to 400 g of freeze-dried material.

### 3.4. Characteristic Data for Natural Compounds

NMR data for Compounds **1**–**6** as previously reported [29,30,32,33,34,35].

Kavaranolide **7**: white amorphous solid; [α]_D_^25^ +14.5 (*c* 0.13, MeOH); ^1^H and ^13^C NMR spectroscopic data in DMSO-*d*_6_ given in Table 1; ESIMS: *m*/*z* 329.1 [M − H]^−^. HRMS (ESI) *m*/*z* 329.1381 [M − H]^−^ (calcd. for C_19_H_21_O_5_ *m*/*z* 329.1389).

### 3.5. Evaluation of Anti-CHIKV Replicon Properties

#### 3.5.1. BHK-CHIKV-NCT Cell Culture

A stable BHK21 cell line (BHK-CHIKV-NCT), described by Pohjala and coworkers [52], was used for studying potential anti-CHIKV activity. The cell line harbors the CHIKV replicon and continuously expresses the selection marker puromycin acetyltransferase and two marker proteins (enhanced green fluorescent protein (EGFP) and *Renilla* luciferase (*Rluc*)) for detecting the inhibition of the viral replicon. The BHK-CHIKV-NCT cells were subcultured three times a week and maintained at 37 °C, 5% CO_2_ and 95% humidity in Dulbecco’s Modified Eagle’s Medium with high glucose and l-glutamine (Gibco^®^) supplemented with 7.5% fetal bovine serum (FBS), 2% tryptose-broth phosphate, 1 mM sodium pyruvate, 100 IU/mL penicillin, 100 μg/mL streptomycin and 5 μg/mL puromycin.

#### 3.5.2. CHIKV Replicon Assay, *Rluc* Detection

BHK-CHIKV-NCT cells were seeded onto opaque-white, clear-bottomed 96-well plates (PerkinElmer Inc., Waltham, MA, USA) with a cell density of 40,000 cells/well. The cells were exposed to test samples after 24 h of incubation at 37 °C. The sample stocks were diluted into assay medium consisting of Dulbecco’s Modified Eagle’s Medium with high glucose and l-glutamine (Gibco^®^) supplemented with 5% fetal bovine serum (FBS), 1 mM sodium pyruvate, 100 IU/mL penicillin and 100 μg/mL streptomycin. Puromycin was excluded from the assay medium to avoid puromycin-induced toxicity. The exposure time to test samples was 48 h, after which the *Rluc* expression was determined by using a *Renilla* luciferase assay kit (Promega, Madison, WI, USA), according to the manufacturer’s instructions. The luminescence signal was recorded using a Varioskan Flash plate reader (Thermo Fischer Scientific, Vantaa, Finland) with a measurement time of 1 s and automatic dynamic range settings. The primary test concentration for crude extracts was 100 μg/mL (*n* = 3). The percentage of inhibition of the viral replicon was calculated by comparing the sample signal to the yielded maximum signal (DMSO vehicle in assay medium), and the activity threshold was set at >50% inhibition of *Rluc* expression.

The screening assay was optimized for *Rluc* detection and validated by dose-response experiments for the positive control, 6-azauridine. The dose-response curve for 6-azauridine determined by *Rluc* detection showed sigmoidal, dose-dependent reduction in the marker level, with an IC_50_ value of 2 μM. The 6-azauridine concentration used as a positive control on every assay plate was 10 μM, which caused an average inhibition of 69%. The signal-to-background ratio (S/B) and signal-to-noise ratio (S/N) were calculated for each plate as a measure of assay quality [53]. DMSO in assay medium represented the maximum signal and wells with only medium and reagents the background.

#### 3.5.3. Cytotoxicity Assay

The viability of BHK-CHIKV-NCT cells was determined after treatment with hit samples in order to exclude possible false positives. The test conditions were identical to those described for the *Rluc* assay. After 48 h of exposure to the samples, cell viability was determined by ATP quantitation using a CellTiter GLO^®^ Luminescent Cell Viability Assay kit (Promega, Madison, WI, USA). Briefly, the cells were equilibrated to room temperature and washed with 100 μL of phosphate buffered saline solution, after which 50 μL of CellTiter GLO^®^ Reagent and 50 μL of assay medium were added. The plate was subjected to shaking for 2 min to induce cell lysis, and after 10 min, the luminescence was measured by using a Varioskan Flash plate reader.

Each sample was tested in triplicate, and the cytotoxic effect of test samples was determined as a percentage by using the maximum signal (DMSO vehicle in the assay medium) as the reference and cells with no reagent added as the background value. The cytotoxicity assay was validated by dose-response experiments for the positive control, polymyxin B, which showed dose-dependent cytotoxicity. The ED_50_ for the cytotoxic activity of polymyxin B was determined as 5900 IU/mL. The polymyxin B concentration used in the experiments was 10,000 IU/mL and caused an average toxicity of 93%.

#### 3.5.4. CHIKV Replicon Assay, Live Cell Imaging

The effects of the control compounds and test samples on proliferation, morphology and expression of EGFP in BHK-CHIKV-NCT cells was analyzed in a continuous cell culturing platform with integrated optics (Cell-IQ^®^ Fluorescence, Chip-Man Technologies Ltd., Tampere, Finland). The expression of EGFP is an equally valid marker as *Rluc* for the inhibition of the CHIKV replicon.

The test conditions were optimized by evaluating different imaging settings and cell densities. The cells were seeded onto clear-bottomed black-framed 96-well plates (PerkinElmer Inc., Waltham, MA, USA) with a density of 3000 cells/well and incubated for 24 h at 37 °C before starting the treatment. The samples were diluted into assay medium, as for the *Rluc* and cytotoxicity assays. Each sample was tested in triplicate. After starting the treatment, phase-contrast and fluorescent images were taken with 30 min intervals for 48 h using a 10× objective. Before starting the imaging, a Cell-Secure Lid (Chip-Man Technologies, Tampere, Finland) equipped with a gas input connector and a sterile 0.2-μm filter was sealed to the plate. The plate was incubated at 37 °C using the manufacturer’s default CO_2_ flow setting (8 min flow, 20 min pause, 30 min initial flow). For the phase contrast images, the *z*-stack was 17.60 μm and the exposure time 10 ms; for the fluorescent images, the default imaging settings for green fluorescent protein with binning 2 × 2 and an exposure time of 200 ms were applied.

Protocols for analyzing the images captured in Cell-IQ^®^ were created according to the manufacturer’s instructions using the Cell-IQ Analyser^®^ software (Chip-Man Technologies, Tampere, Finland). Details on the creation of analysis protocols can be found in the Supplementary Information.

**Figure 6:**
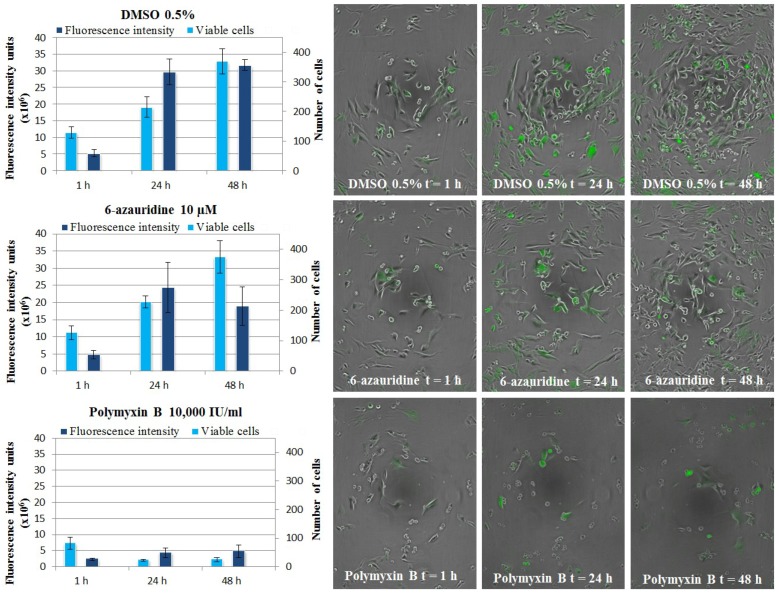
The effects of control compounds (DMSO vehicle, 6-azauridine, polymyxin B) on BHK-CHIKV-NCT cells imaged with Cell-IQ^®^ and analyzed with Cell-IQ Analyser^®^ software: Fluorescence and cell count at 1 h, 24 h and 48 h and combined fluorescent and phase contrast images for each time point. The number of viable cells is determined as the sum of cells classified as living and dividing.

The experiments performed in Cell-IQ^®^ were validated through tests on the control compounds used in the *Rluc* assay and the cytotoxicity assay: DMSO vehicle in the assay medium, 6-azauridine as a model antiviral agent and polymyxin B as a model cytotoxic agent. BHK-CHIKV-NCT cells treated with control compounds were imaged for 48 h, after which the created analysis protocols were applied. The results of cell classification and fluorescence intensity analysis for BHK-CHIKV-NCT cells treated with control compounds are shown for three time points in Figure 6. Positive control 6-azauridine decreases the fluorescence intensity of the cells, without affecting cell morphology, whereas polymyxin B radically decreases both the number of viable cells and, as a consequence, the fluorescence intensity.

Percentages of CHIKV replicon inhibition based on fluorescence detection and cytotoxicity based on cell classification were calculated for each sample. To additionally increase the reliability, results reported for test samples are averages from two independent assays with 3 replicate wells each.

### 3.6. Evaluation of Anti-Inflammatory Properties

#### 3.6.1. Preparation of Microglial Primary Cell Cultures

Primary microglial cell cultures were prepared from brains of newborn Wistar rats [54]. Ethics approval for the experiments was granted by the EBIOTEC Review Board (Valter Lombardi, Iván Carrera and Lucía Fernández-Novoa; Project Identification Code: Sinularia Project October 2013; approved 30 October 2013). The neocortex of one-day-old postnatal rats was aseptically dissected out, and the meninges and blood vessels were carefully removed. Following cutting in a medium containing 20% horse serum, the suspension was filtered through a 40-mm filter and transferred as a single cell suspension to culture dishes, using 5.0 mL of medium per dish. The cultures were incubated at 37 °C in an atmosphere containing 5% CO_2_. The culture medium consisted of Minimum Essential Medium Eagle, supplemented with l-glutamine, amino acids, vitamins, penicillin, gentamicin, sodium bicarbonate and 20% heat-inactivated horse serum. The medium was changed twice a week. After 1 week, the concentration of horse serum was reduced to 10%. After sixteen days in culture, cells were treated with 1 μg/mL of lipopolysaccharide (LPS). After six hours, different concentrations (1.0 μg/mL, 2.5 μg/mL and 5.0 μg/mL) of Compounds **1**–**4** and **6** were added to each well, and plates were incubated for 72 h at 37 °C in an atmosphere containing 5% CO_2_. Positive controls were treated only with LPS, while negative controls were treated with complete medium. After 72 h of incubation, cells were counted, and 2.5 mL of the supernatants were collected from each well and frozen at −40 °C for cytokine analyses. All experiments were carried out twice.

#### 3.6.2. Cell Count

Statistical analysis was carried out from three randomly chosen untreated dishes and three randomly chosen treated dishes. Microglial density values were obtained by counting 10 fields in the microglial area for each cell culture using a 20× objective. The ANOVA test was performed at the 95% significance level in order to contrast differences.

#### 3.6.3. Determination of Cytokine Concentration

The supernatants obtained from cell cultures were analyzed for TNF-α, IL-1β, IL-4, IL-6, IL-8, IL-10 and IL-18 using commercially available ELISA kits following the manufacturers’ instructions. All samples were assayed in duplicate, and equivocal results were repeated. The cytokine concentration was calculated from a standard curve of the corresponding recombinant cytokine.

#### 3.6.4. Determination of IC_50_ Values by Quantitation of Cytokine Release and Colorimetric Methyl Thiazol Tetrazolium (MTT) Cytotoxicity Assay

The cytotoxicity of the compounds was measured by the colorimetric methyl thiazol tetrazolium (MTT) assay and scored as a percentage of the absorbance reduction of treated cultures *versus* untreated control cultures. Primary microglial cell cultures were seeded into 96-well microplates at 10^4^ cells per well and allowed to grow for 24 h. The initial concentration of Compounds **1**–**4** and **6** was 100 μg/mL in DMSO. The compounds were serially diluted in complete culture medium with two-fold dilutions (100, 50, 25, 12.5, 6.25, 3.12 and 1.56 μg/mL for *in vitro* cytotoxicity; 12.5, 6.25, 3.12, 1.56, 0.78 and 0.39 μg/mL for cytokine release). Different concentrations of the compounds were added to each well, and Tamoxifen was used as a positive control, with concentrations ranging from 50 to 1.56 μg/mL. Plates were incubated at 37 °C for 72 h under 5% CO_2_ atmosphere. Then, the 50 μL of MTT-PBS solution in culture medium were added to each well. The plates were further incubated for 4 h under the same conditions. The medium was then removed and replaced with 200 μL of DMSO to solubilize the MTT formazan product. The solutions were shaken for 20 min, and the absorbance was measured at 570 nm. One hundred microliters of the supernatants collected after incubation were used for cytokine quantification, as described in Section 3.6.3, and prednisolone was used as a positive control. Each value represents the mean of triplicate determinations. The IC_50_ values were calculated from the compound concentration-response curves.

#### 3.6.5. Statistics

The results are expressed as the mean ± standard deviation. Paired and unpaired Student’s *t*-tests were used to determine the significance of differences; a value of *p* < 0.05 was considered statistically significant.

## 4. Conclusions

The investigation of bioactive natural products from an Indian soft coral, *Sinularia kavarattiensis*, led to the isolation of a novel norcembranoid, named kavaranolide (**7**), along with six known compounds (**1**–**6**). Despite its inactivity in the CHIKV replicon model, the isolation and characterization of kavaranolide (**7**) adds value to the outcomes of this study. Imaging experiments on the CHIKV replicon model with the isolated compounds indicated that both **1** and **2** show moderate activity against the CHIKV replicon, but also remarkable cytotoxic properties. The effects of the isolated compounds on primary microglial cells, on the contrary, clearly indicate that Compound **2** is able to regulate the morphology and function of surveying/resting microglia and to decrease the activation of microglia, which could contribute to the progression of chronic neurodegenerative diseases. Since Compound **2** also shows potential anti-inflammatory properties based on the effect on cytokine release, it is possible to hypothesize that it may have the capacity to reduce the release of a large number of substances that can be detrimental to the surrounding neurons and finally contribute to a CNS homeostasis. In recent years, several natural products isolated from herbal plants [55] were proven to act as inhibitors of microglial neurotoxicity. This study represents the first report of a marine natural product with modulatory activity on neuroinflammation.

## Supplementary Files

Supplementary File 1 Supplementary Information (PDF, 225 KB)
