# Supplementary material for: Bioactive Cembrane Derivatives from the Indian Ocean Soft Coral, Sinularia kavarattiensis
Source: Mar Drugs. 2014 Jul 3;12(7):4045–68. doi: 10.3390/md12074045 (PMC4113814; doi:10.3390/md12074045)
Supplement: Supplementary File 1 — Supplementary Information (PDF, 225 KB) [file marinedrugs-12-04045-s001.pdf]

Supplementary Information

S1. Building the Analysis Protocols in Cell-IQ Analyser®

Protocols for analyzing the images captured in Cell-IQ® were created according to the manufacturer's instructions using the Cell-IQ Analyser® software (Chip-Man Technologies, Tampere, Finland). Two separate protocols were built: one for cell counting based on morphology analysis and another for fluorescence intensity measurement. The cell counting protocol was built by first setting segmentation parameters, such as maximum cell diameter and symmetry, the distance between cells and the segmentation gradient threshold. Second, a sample library for the recognition of different cell morphologies was built by teaching the software to classify recognized cells as living healthy cells, dead cells and dividing cells (Figure S1). Additionally, classes for background and cell debris were created to avoid false recognitions.

Figure S1. Sample library for image analysis created in Cell-IQ Analyser®: Cell classification images for cell classes living, dead and dividing.

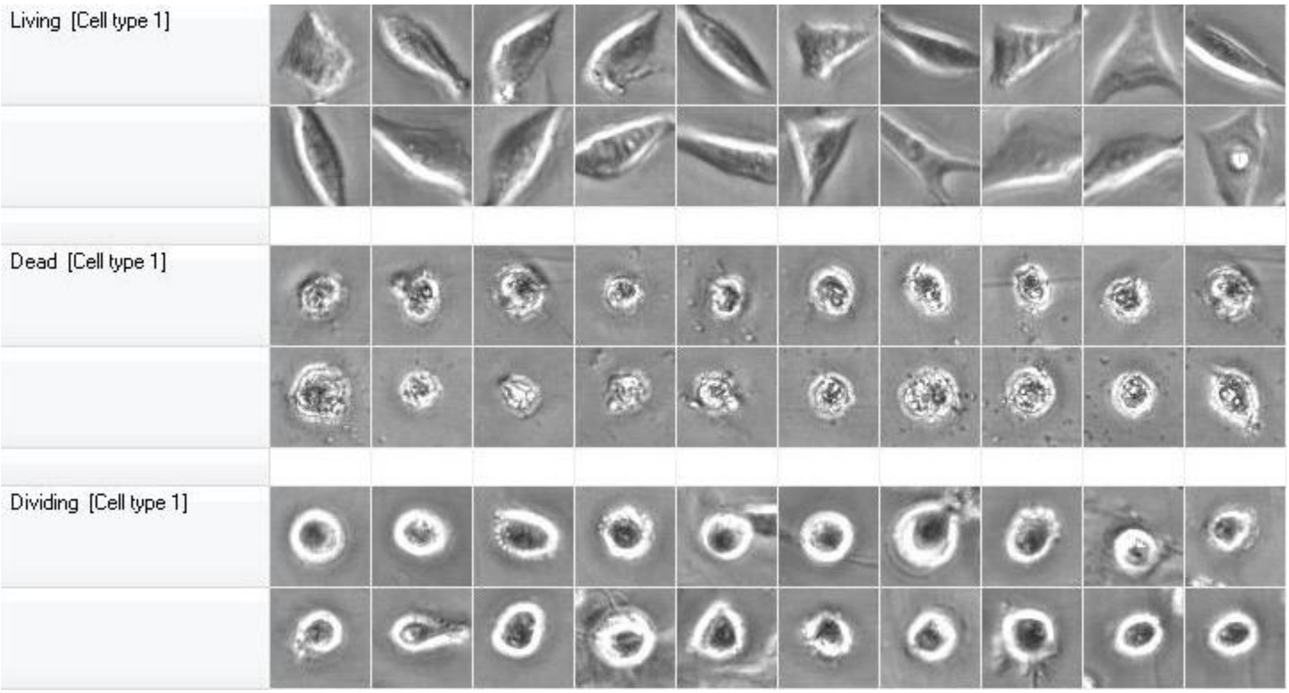

The cell counting protocol was optimized by analyzing different image sets and evaluating the classification accuracy, followed by changes to analysis parameters or the modification of the sample library. An example image to which the classification protocol has been applied is shown in Figure S2 to illustrate the analysis.

Figure S2. Classification of BHK-CHIKV-NCT cells with Cell-IQ Analyser® (DMSO 0.5%, *t* = 48 h). Classification: red = living cells; green = dead cells; blue = dividing cells.

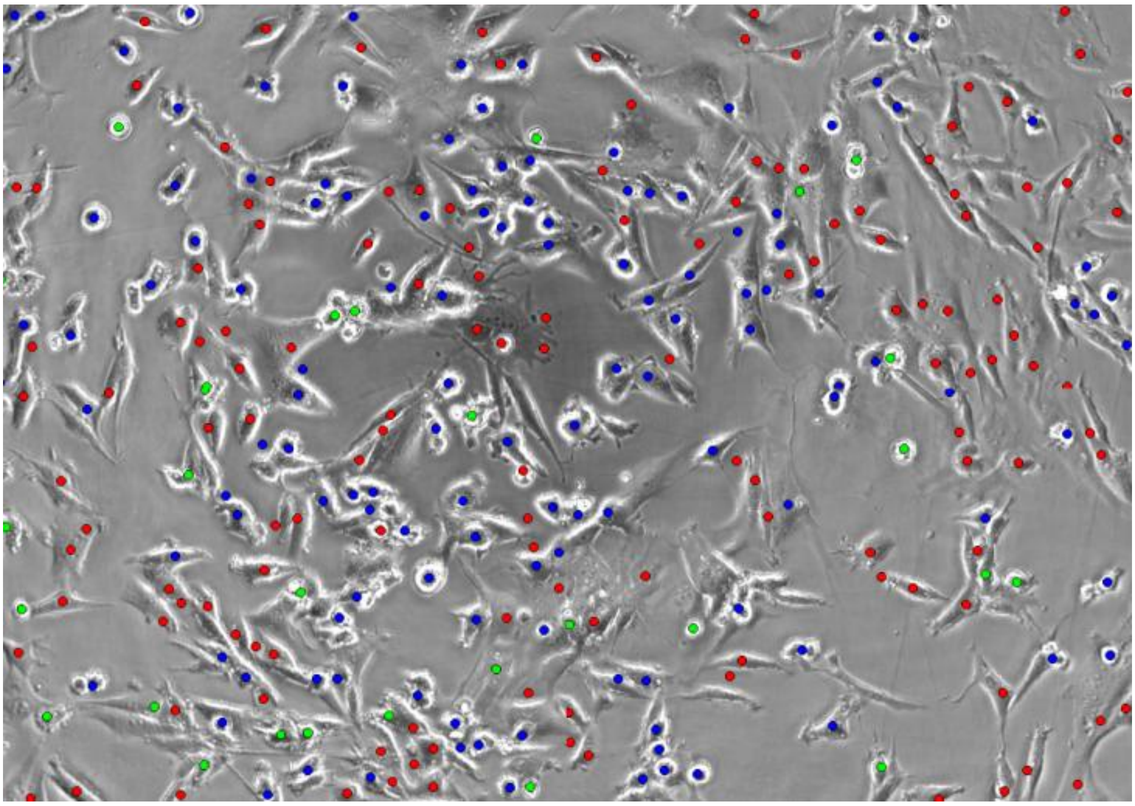

The fluorescence intensity measurement protocol was built by optimizing the analysis parameters for brightness, contrast, gamma, background correction and fluorescence threshold. The software determines the fluorescence cell count, area and intensity for each captured image and allows seeing the effects of changes to the analysis parameters in preview images. The optimized settings were tested for a sample set of fluorescence images to ensure accuracy.

The experiments performed in Cell-IQ® were validated through tests on the control compounds used in the *Rluc* assay and the cytotoxicity assay: DMSO vehicle in assay medium, 6-azauridine as a model antiviral agent and polymyxin B as a model cytotoxic agent. The effect of control compounds on fluorescence intensity throughout the 48 h experiment is shown in Figure S3. Finally, the optimized protocols were used to analyze the image sets captured during experiments.

Figure S3. The effect of control compounds (measurement time 48 h) on the fluorescence intensity of BHK-CHIKV-NCT cells in Cell-IQ® live cell imaging. The results are the averages of three independent tests with three replicates each.

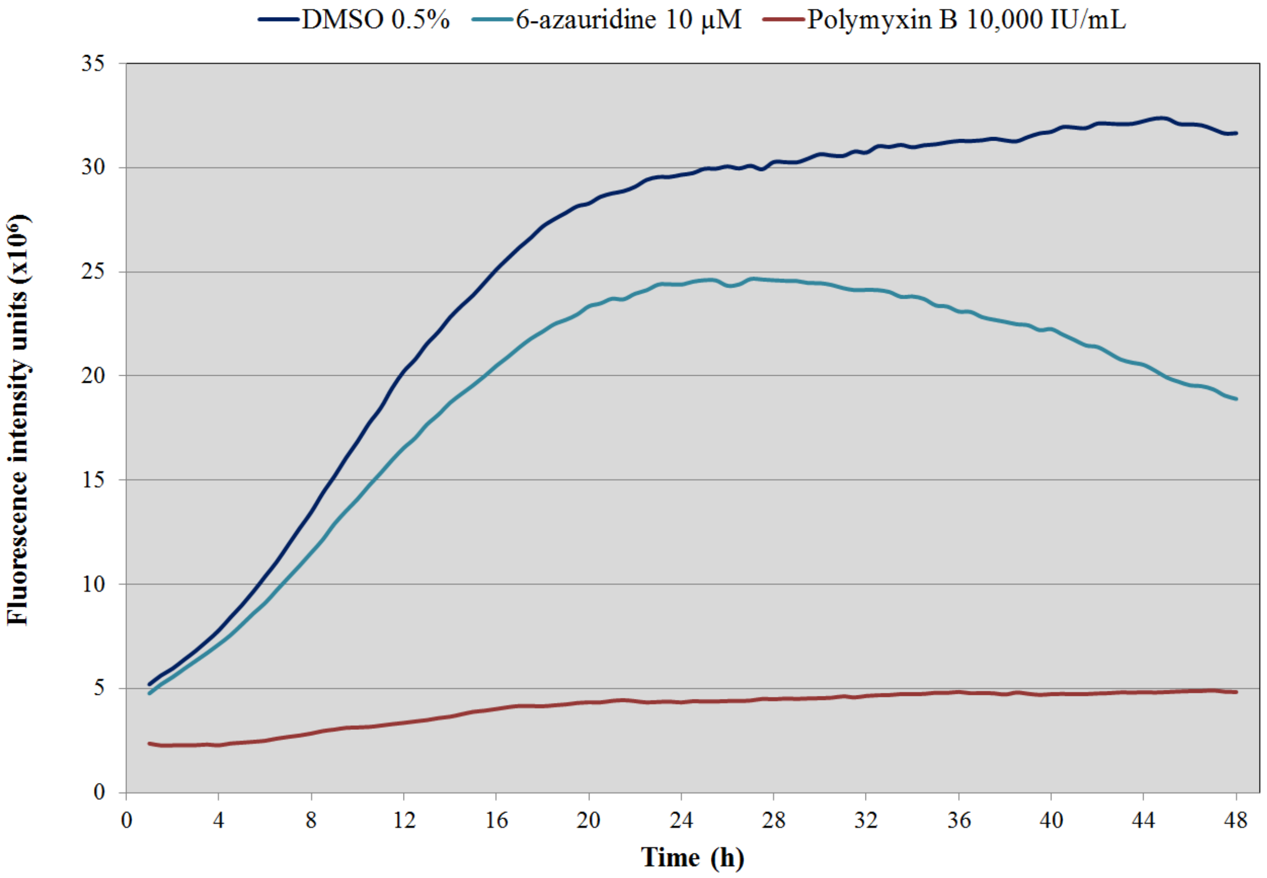

© 2014 by the authors; licensee MDPI, Basel, Switzerland. This article is an open access article distributed under the terms and conditions of the Creative Commons Attribution license (http://creativecommons.org/licenses/by/3.0/).
